# Spotlight on Human LL-37, an Immunomodulatory Peptide with Promising Cell-Penetrating Properties

**DOI:** 10.3390/ph3113435

**Published:** 2010-11-01

**Authors:** Michèle Seil, Carole Nagant, Jean-Paul Dehaye, Michel Vandenbranden, Marc Ferdinand Lensink

**Affiliations:** 1 Laboratoire de Chimie Biologique et Médicale et de Microbiologie Pharmaceutique, Institut de Pharmacie, Université Libre de Bruxelles, Boulevard du Triomphe, CP 205/3, B-1050 Brussels, Belgium; 2 Laboratoire de Structure et Fonction des Membranes Biologiques, Faculté des Sciences, Université Libre de Bruxelles, Boulevard du Triomphe, CP 206/2, B-1050 Brussels, Belgium; 3 Biological Nanosystems, Interdisciplinary Research Institute, University of Sciences and Technology Lille, USR3078 CNRS, 50 Avenue Halley, F-59658 Villeneuve d’Ascq, France

**Keywords:** antimicrobial peptides, biofilm, P2X_7_ receptors, formyl peptide receptors, cell-penetrating peptides, LL-37, cathelicidin

## Abstract

Cationic antimicrobial peptides are major components of innate immunity and help control the initial steps of the infectious process. They are expressed not only by immunocytes, but also by epithelial cells. They share an amphipathic secondary structure with a polar cationic site, which explains their tropism for prokaryote membranes and their hydrophobic site contributing to the destructuration of these membranes. LL-37 is the only cationic antimicrobial peptide derived from human cathelicidin. LL-37 can also cross the plasma membrane of eukaryotic cells, probably through special domains of this membrane called lipid rafts. This transfer could be beneficial in the context of vaccination: the activation of intracellular toll-like receptors by a complex formed between CpG oligonucleotides and LL-37 could conceivably play a major role in the building of a cellular immunity involving NK cells.

## 1. Introduction

Epithelia are constantly exposed to potential pathogens. The digestive tract, the upper respiratory airways, the genital tract and the skin are, among others, body surfaces interacting with the surroundings and very often a door to aggression by a pathogen. The response of adaptative immunity to this aggression is highly efficient and very often successful in fighting an infection, but adaptation is slow and linked to delay. In a first step, cells producing membrane-anchored antibodies with reasonable affinity to the antigen have to be selected. In a second step, these antibodies are secreted in the extracellular medium. The tremendous dilution of these proteins decreases their concentration to extremely low values, which jeopardizes their interaction with the antigen. The dilution must be compensated by an increase of the affinity of the protein for the antigen. This affinity maturation is the consequence of a very high mutational rate of the gene, a high proliferation rate of the cells and a very strong selective pressure. This process is considered a paradigm for evolution, but at a timescale of a few days rather than a few million years [1]. A similar selection and maturation process forT lymphocytes contributes to the delay of a strong and specific attack of the pathogens. During the building and shaping of this adaptative immunity, the pathogens would have plenty of time to multiply and would reach, after a few days, such a number that they would provoke the death of the invaded body. This would render adaptative immunity a very elegant but futile response to the lethal aggression. The innate immunity prevents this fatal issue. The mechanisms of innate immunity are designed to block or at least to delay the progression of the infection. This very rapid response is triggered by the recognition by specialized cells like antigen-presenting cells of unusual molecular patterns which are specifically associated with pathogens. Among the various responses elicited by local infections, antimicrobial peptides have generated great interest since their discovery by the group of Boman in 1980 [2]. During these last three decades the number of antimicrobial peptides has been constantly increasing and by October 2010 the antimicrobial Peptide Database created by Wang and Wang [3] contained 1,628 references. These peptides are designed to kill bacteria, viruses and fungi. They interact with their targets in a rather non-specific way which explains why the pathogens have some difficulty to elaborate mechanisms of resistance against these peptides. More recently new modes of action of these peptides have been discovered and at concentrations which are bactericidal they also activate eukaryotic cells. Among other responses they contribute to the mobilization of immunocytes at the site of aggression. These peptides originally described as antimicrobials are now considered as “alarmins” [4]. In mammals two main families of antimicrobial peptides have been described: the defensins [5] and the cathelicidins [6]. Other peptides with antimicrobial properties have also been described in humans. For instance histatins are salivary peptides rich in histidine residues and which have a strong fungicidal activity [7]. Neuropeptides like VIP have also microbicidal properties [8]. Lactoferricin is a cationic peptide which is derived from lactoferrin. It is present in mucosal secretions and in milk [9]. Dermicidin is secreted as a precursor by sweat glands and is activated by cathepsin D [10]. Some antimicrobial peptides are derived from proteins which have apparently no obvious role in innate immunity as the amyloid beta protein associated with Alzheimer’s Disease [11], proenkephalin-A [12], apolipoproteins A, B or E [13,14,15], growth factors [16] or members of the complement system or superoxide dismutase [17]. After a brief description of defensins and cathelicidins, this review will focus mostly on LL-37, the only human antimicrobial peptide derived from cathelicidin.

## 2. Defensins

Defensins are small (approximately 4 kDa), cysteine-rich, cationic peptides which have been identified in plants, insects, and a large variety of higher-level mammals [5]. They have been classified in three distinct families: α-, β- and θ-defensins. The θ-defensins are circular peptides derived fromα-defensins. DEFT, the gene for θ-defensins, probably arose from duplication of the gene forα-defensin in Old World apes. It mutated after divergence of the orangutan and hominid lineages. These mutations introduced stop codons in the open reading frame and suppressed the synthesis of the peptide [18]. θ-Defensin is thus not expressed in man and it has been suggested that the absence of this defensin is responsible for our sensitivity to HIV infection [18]. The two other families of defensins are expressed in man. These peptides contain six cysteine residues forming three disulfide bridges. These bridges not only play a role in the structure of the peptides but also contribute to their resistance to intracellular and extracellular proteases [19,20]. The spacing between the cysteine residues and the topology of the disulfide bonds is the rationale for the distinction of these peptides in two families, the α-defensins and the β-defensins [21]. 

The genes for α- and β-defensins are located on chromosome 8. They form a cluster within a < 1 Mb region at the locus p22-23 [22]. Defensins are produced as immature peptides, the pre-prodefensins, that contain a signal sequence at the *N*-terminus, followed by a propeptide sequence, and the mature defensin peptide at the C-terminus [23]. After the cleavage of the signal peptide the pro-defensin is released in the endoplasmic reticulum. The α-prodefensin has an *N*-terminal sequence of about 40 amino acids. This prosequence is anionic which probably accounts for the binding of the mature defensin to the propeptide. α-Prodefensin acts as a chaperone and contributes to the structure of mature defensin. It also blocks the activity of the mature defensin preventing damages to the producing cells [24]. In man, the α-prodefensin has no microbicidal activity and its activation requires its proteolysis by serine proteases like trypsin [25], kallikrein [26] or metalloproteases [27]. The α-defensins have 29-35 amino acids. Four isoforms are found in granules of neutrophils and two isoforms are expressed in Paneth cells of the small intestine [28]. The β-defensins are also synthetized as prepropeptides. The *N*-terminal extension of the proform has a very short amino acid sequence; the proform has bactericidal activity [27] and even some cellular toxicity [29]. The mature peptides have~ 40 residues. At least six isoforms have been isolated [30]. They are expressed in epithelia, the testis and the epididymis [31]. Both α- and β-defensins share a common motif termed the γ-core. It is composed of two antiparallel β-sheets with basic residues polarized along its axis [32]. 

## 3. Cathelicidins

**Figure 1:**
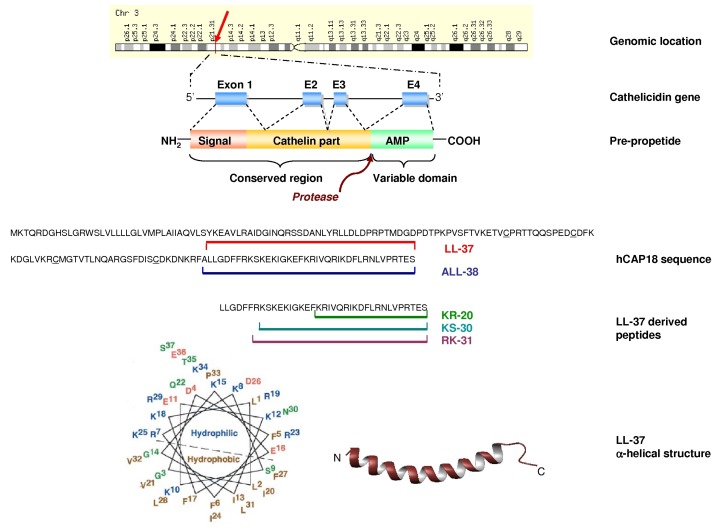
Structure of LL-37. From top to bottom: Location of the cathelicidin gene on the human genome and its structure. Global structure of the pre-propeptide and primary structure of hCAP-18. Sequence of various fragments of LL-37 and model representing the secondary alpha-helicoidal structure of LL-37 [94].

The first antimicrobial peptide of this group was isolated from pig [33]. The term cathelicidins was introduced in 1995 by Zanetti *et al.* [34] to describe molecules containing both a cathelin domain and a C-terminal antimicrobial domain. Cathelin is an acronym for cathepsin L inhibitor. The human cathelicidin has 18 kDa (hCAP-18) and is a major protein in specific granules of neutrophils [35]. It is also present in subpopulations of lymphocytes and monocytes, in squamous epithelia, in epididymis [36] and in the lung [37,38]. Several resident cells of the skin like keratinocytes, mast cells or sebocytes also express hCAP-18 [39,40,41]. Plasma contains a high concentration of hCAP-18 bound to lipoproteins [42]. The pre-proregion of cathelicidins has 128-145 residues: a signal peptide with 29-30 residues and a proregion with 99-114 residues (Figure 1). This proregion shows a high intra-species identity ranging from 75 to 100% homologies between species. Four invariant cysteinyl residues in the C-terminal region of the cathelin-like domain form two intramolecular disulfide bridges. 

Considering the conservation of this proregion during evolution, it might play an important biological function with respect to the maturation of the antimicrobial peptide which is the *C*-terminal domain of the protein. Zaiou *et al.* [43] reported that the cathelin domain had also potent antibacterial activity. The *C*-terminal domain of cathelicidin is very variable among species. In man, the only peptide derived from hCAP has 37 amino acids and two leucines at its *N*-terminal, hence the acronym LL-37 [44]. The human gene coding for hCAP-18 is named CAMP. This gene is located on chromosome 3 at the p21 locus [45], a locus frequently deleted in gastric carcinoma [46]. This might explain why the concentration of the peptide is reduced in human gastric adenocarcinomas [47,48]. The full peptide was first isolated from bone marrow [49,50], then from the secretions of neutrophils [49]. The release of LL-37 from its precursor is mediated by proteinase 3 [51] or elastase [49]. In vagina, gastricsin, a prostatic protease, releases a peptide with 38 amino acids, ALL-38 [52]. In human sweat the mature LL-37 has been shown to be degraded to shorter peptic fragments, including RK-31, KS-30 and KR-20 [53]. These fragments are generated by two distinct kallikreins [54]. These natural and more recently synthetic fragments of LL-37 have contributed to a better understanding of the full peptide. LL-37 has broad bactericidal activity toward both Gram-negative and Gram-positive bacteria [55]. It neutralizes LPS, it has synergistic antibacterial effects with the defensins [56] and it is a chemotactic agent for neutrophils, monocytes and T cells using the formyl peptide receptor-like 1 (FPRL-1) [57]. It also interacts with a wide panel of other plasma membrane receptors and has access to some intracellular compartments of eukaryotic cells. However, LL-37 is also cytotoxic towards mammalian cells [58]. 

## 4. Effects of LL-37 on Prokaryotes

Patients with atopic dermatitis suffer not only from chronic cutaneous inflammation but are also affected by recurrent infections provoked by bacteria, viruses or fungi [59]. The skin of these patients is characterized by a decreased expression of defensins and LL-37, suggesting a role for these peptides in skin protection [60]. Drugs increasing the local concentration of antimicrobial peptides have thus been logically proposed as a treatment for atopic dermatitis [61]. The role of antimicrobial peptides is fully supported by experiments with transgenic animals. *Salmonella typhimurium* survive better in macrophages from mice which do not express the cathelicidin related antimicrobial peptide (CRAMP), the murine analog of LL-37, than from wild-type (WT) mice [62]. These mice are also more prone to infections of the skin by *Staphylococcus aureus* [40] or to meningococcal infections of the central nervous system [63] and to infections of the urinary tract [64]. Conversely, Bals *et al.* [65] demonstrated that mice overexpressing LL-37 had a lower bacterial load and reduced inflammatory response in the lung after a challenge with *Pseudomonas aeruginosa*. They also showed that the transfer of the LL-37/hCAP18 gene restored bacterial (*Pseudomonas aeruginosa* and *Staphylococcus aureus*) killing in a human cystic fibrosis bronchial xenograft model [66]. Cathelicidin is highly expressed at barrier sites including respiratory and colonic epithelium, saliva, and skin. At these locations it constitutes an important first line defense mechanism for the innate immune system to respond to infectious agressions [67]. It is active in the micromolar concentration range, with a higher tropism for Gram-negative than for Gram-positive bacteria [68].

### 4.1. Structure of LL-37 and Biophysical Studies

At the structural level, efforts were made to understand how LL-37 and related peptides interact with the membrane and what type of perturbation they may cause. To have access to the molecular level, simplified systems were used, comprising phospholipid bilayers made of a limited series of commonly found phospholipids. Since cathelicidins have a positive balance of cationic amino acid side-chains, the focus was put on the possible difference between zwitterionic and acidic phospholipid-containing membranes.

The interpretation of initial circular dichroic studies on the peptide diluted in pure water led to conclude that LL-37 adopted a random conformation in aqueous solution. It became rapidly obvious that the peptide could structure itself either in the presence of the salts found in physiological fluids [58] or at higher concentrations of the peptide which seemed to favour structuring oligomerization as demonstrated by chemical cross-linking experiments [69]. In the presence of a phospholipid bilayer or short-tail phospholipid micelles, LL-37 preferentially adopts an alpha-helical conformation as shown by FTIR [69,70], circular dichroism [70,71,72,73] and NMR [73,74,75,76]. The mean orientation of the helix is closely parallel to the bilayer surface. This was shown independently by solid-state NMR on macroscopically aligned DMPC bilayer samples where the helical part of the structure forms an angle of about 72° with the bilayer normal [73] and by polarized ATR-FTIR spectroscopy on oriented bilayers [69,70]. As the alpha-helix is amphipatic, it is expected to burry its hydrophobic residues in the hydrophobic part of the bilayer while the polar/charged residues should stay at the level of the lipid polar headgroups. This topology is consistent with NMR data [69,74]. How this topology could explain the toxic effect of cathelicidins is less clear (see next paragraph on the mode of action of LL-37 on prokaryotes). The weak point of the spectroscopic techniques used to determine the peptide topology results from their inability to detect minor but perhaps significant subpopulations of other topological arrangements that would be responsible for the membrane-destabilizing or lytic activity. Furthermore, the main question of course is to evaluate the role of a direct membrane-destabilizing effect over a more indirect effect that relies on the activation of some cell signalling cascades, possibly via membrane receptors (see the section on the effects of LL-37 on eukaryotic cells). Experimental results on liposomes do support the existence of a direct permeabilizing effect on phospholipid bilayers [69,74,77], but only a few lipid compositions were used and therefore the role of some specific membrane lipids might have been overlooked. The question of a selective destabilizing effect of cathelicidins towards bacterial *versus* eukaryotic membranes was partly addressed at the lipid level, using lipids present in both types of organisms, but which are not fully exposed to the outer membrane leaflet, such as the acidic phospholipid phosphatidylserine (PS), phosphatidylglycerol (PG) and the non-bilayer forming unsaturated phosphatidylethanolamine (PE), the latter being abundant in prokaryotes. Whereas it was initially demonstrated that similar leakage occured in zwitterionic palmitoyl-oleoyl-phosphatidyl-choline (POPC) vesicles as well as in charged palmitoyl-oleoyl-phosphatidyl serine/palmitoyl-oleoyl-phosphatidylcholine (POPS/POPC) vesicles when considering K^+^ permeabilization [69], further studies demonstrated a preference for negatively charged vesicles composed of palmitoyl-oleoyl-phosphatidylglycerol (POPG) as compared to neutral zwitterionic POPC vesicles when larger molecules such as calcein were considered [71]. In another study, a mixture of neutral lipids (PC/sphingomyelin/cholesterol) (PC:SM:CHOL) seemed equally susceptible as acidic lipids phosphatidylglycerol/diphosphatidylglycerol towards a leakage assay of a mixture of 8-amino- naphthalene-1,3,6-trisulfonic acid/*p*-xylene-bis-pyridinium bromide (ANTS/DPX) with a slight preference for the PC:SM:CHOL mixture at low peptide concentration, especially with the orang-utan orthologue of the human LL-37 [70].

In accordance with typical antimicrobial peptide behavior, cholesterol diminished LL-37 induced leakage in the study of Zhang *et al.* [71], whereas it has no such effect in the study of Morgera *et al.* [70]. From theses results and others, it seems difficult at present to confirm any clear lipid preference for LL-37 that would explain a selective effect on bacteria over mammalian cells [78]. Clearly, other features of the membrane lipid composition should be taken into account, such as the presence of LPS or peptidoglycans in the bacterial wall or complex glucosaminoglycans in the case of mammalian cells and perhaps the transmembrane electrical potential. Other parameters than membrane leakage could play a significant role such as lipid clustering [79] or membrane thickening effects [80]. Many studies failed to show a clear discrimination in the toxic effect on prokaryotes and eukaryotes, although some LL-37 orthologues seem generally at least an order of magnitude more effective towards bacteria [72]. Demonstration of such an effect is complicated by the way toxicity is measured in both cases. Indeed, while the focus is put on the peptide concentration, its ratio to the total lipid mass may vary considerably, bacteria being usually tested at a lower cell concentration [81].

### 4.2. Mode of Action of LL-37 on Prokaryotes

LL-37 is a peptide with 35% hydrophobic residues. It has a high content of basic residues(five arginines; six lysines) and at neutral pH it has a positive charge (+6). In spite of their very diverse primary structures, antimicrobial peptides seem to share some common structural characteristics (see previous paragraph on the structure of LL-37 and biophysical studies). They all form amphipathic secondary structures with a cationic and a hydrophobic face [82,83]. This characteristic seems mandatory for their interaction with the bacterial membranes [84,85,70]. Three mechanisms have been suggested for the permeation of the target cell membrane by antibacterial peptides. A barrel stave mechanism involves the formation of transmembrane channels in a voltage-dependent manner with nonpolar domains of the molecules facing the membrane lipids and forming a hydrophilic pore spanning the membrane [86]. In the aggregate channel model, intracellular components leak out of the cells through transient pores formed by peptides in unstructured clusters in the membrane. In the carpet-like mechanism the antimicrobial peptides cover the cell membrane. The bending of the lipid bilayer on itself forms holes and destroys the integrity of the membrane [87]. LL-37 binds to bacterial membrane [88]. At high pH or in the presence of detergents or lipids LL-37 forms an alpha-helix covering residues 2 to 31 [58,69,89,90] with a cationic and a hydrophobic side. The formation of this helix increases the rigidity of the entire molecule and correlates with its lytic potency [70]. Only residues 33 to 37 are mobile and do not contribute to the interaction with the membrane [91]. These authors also showed that the interaction with a membrane stabilizes the alpha-helix formed by LL-37. Oren *et al.* [69] have suggested that LL-37 permeabilizes the bacterial membrane by a carpet-like mechanism. LL-37 is able to aggregate and to form a tetramer [92]. KR-20, the fragment of LL-37 after removal of the 17 residues from the *N*-terminal is still able to aggregate. In contrast, deletion of the 14 *C*-terminal residues greatly reduces the aggregation of LL-23 [92]. KE-18 also forms an alpha-helix [50,93] and inhibits the interaction between LPS with CD14 [94]. When LL-37 is cut into two fragments, only the *C*-terminal fragment (IG-25) retains the toxic activity on bacteria and eukaryotic cells [91]. The same group also showed that removal of residues not essential for the interaction of this peptide with micelles generates FK-16, the fragment of LL-37 running from F17 to V32. This fragment has an even better activity against prokaryotes and nucleated cells than the full peptide or than IG-25. Removal of the *C*-terminal tripeptide from FK-16 yields FK-13, a peptide nearly devoid of any activity on bacteria and eukaryotic cells. KR-12 has three arginines and two lysines. It covers the cationic rich region of LL-37. This peptide is the smallest fragment of LL-37 that is still active against Gram-negative bacteria [90]. A longer fragment, GF-17 has antibacterial activity against both Gram-positive and Gram-negative bacteria [95]. This difference is best explained by different interactions with membrane lipids. KR-12 functions by separating the anionic and zwitterionic lipids in the membrane of Gram-negative bacteria. This lateral-phase separation is impossible in Gram-positive bacteria which have mostly anionic lipids. RI-10, the fragment obtained after removal of the amino acid at both ends of KR-12 has no activity [90]. The longer fragment (GF-17) can not only promote the lateral-phase separation but also the permeabilization of anionic membranes explaining why it is active on both types of bacteria. LL-37 has lost the ability to induce lipid segregation probably because of the rigidity of its alpha-helix. The formation of intramolecular ionic interactions might also prevent cationic residues to interact with anionic lipids. 

### 4.3. Interaction of LL-37 Derivatives with Lipid Bilayers

As discussed in the previous section, the mode of action of LL-37 requires the peptide to adopt an amphipathic helix conformation. However, shortened versions like KR-12, FK-16, and especially KR-20, are not amphipathic *per se*, and may resemble classical cell-penetrating peptides in both structure and mode of action. These peptides, with the 16-residue penetratin as their prototypical representative, have been the subject of many studies over the past decade [96] and are suggested to have a common mechanism of action [97]. Despite continued effort, the precise mechanism of internalization remains at present unknown. A receptor and endocytosis-independent pathway is hypothesized [98] with peptide-lipid interactions governing the translocation mechanism [99]. For pep-1, a cell-penetrating peptide with particularly high uptake efficiency rates, initial peptide adsorption was shown to be a crucial factor in the translocation mechanism [100]. Molecular dynamics simulations of penetratin have shown this association to be a fast process, driven by electrostatic interactions, with the presence of negatively charged lipids significantly enhancing binding of the peptide to the membrane [101]; binding to phospholipids was also shown to be tight for CADY [102], a secondary amphipathic peptide used for delivery of siRNA [103]. Surprisingly perhaps, NMR studies of several homeodomain-derived cell-penetrating peptides in membrane-mimicking SDS micelles show uptake efficiency to be highest for the least deeply inserted peptide [104], although this does confirm the importance of electrostatic surface interactions. It is now commonly accepted that cell-penetrating peptides destabilize the lipid bilayer and that this destabilization is due to specific peptide-lipid interactions. This may lead to the formation of aqueous toroidal pores [105]. Adsorption of penetratin on zwitterionic lipid bilayers, as modelled by molecular dynamics simulation, shows the peptide to introduce surface curvature, leading to the formation of lipid vesicles that encapsulate the protein [106]. However, these simulations were performed in the absence of negatively charged phospholipids and may lead to an alternative translocation mechanism involving a non-lamellar lipid morphology [107]. Investigation of the role of lipid composition on membrane perturbation confirms the capacity to increase membrane curvature and form lipid vesicles, but also demonstrates a remodelling of the membrane [97] and a general ability to induce phospholipid domain separation [98]. The role of electrostatics is evidenced by the importance of negatively charged lipids [100,101,108] and their interaction with the cationic peptides [105,109] and in fact hydrophobic forces seem to play no major role in binding [110]. The free energy cost of insertion of a single penetratin helix into a DPPC bilayer was estimated to be about 75 kJ/mol [106], making spontaneous diffusion across the bilayer a prohibitively slow process, but local charge matching and pairing of the ion charges between peptide and lipids show an important improvement of the theoretical transfer energies [111]. The formation of a peptide-lipid structure was also proposed for the energy-independent translocation of pep-1 [99]. The key of the mechanism of action of cell-penetrating peptides is then to lie in their abundance of cationic residues, where arginine seems to favour internalization over lysine [110,111,112]. Although membrane pore formation remains a controversial issue [99], solid-state NMR measurements provide direct evidence for toroidal pores in the action of PG-1, an arginine-rich antimicrobial peptide [108], with the driving force for this formation being the complexation between the arginine guanidinium and lipid phosphate functional groups [113]. Although arginine-phosphate interactions play a stronger role in the lipid-assisted translocation of cell-penetrating peptides *versus* lysine [109], they are not required for efficient internalization [112]. Nevertheless, both uptake efficiency and bilayer destabilization are directly related to arginine content [111,114]. The stronger interaction with lipid phosphate groups of arginine over lysine results in a more efficient destabilization of the lipid bilayer, a condition that seems to be required for any of the proposed translocation mechanisms [114]. Complexation of arginine and phosphate combined with bilayer undulations allow the insertion of charged side chains in the bilayer core that nucleate the formation of a transient, short-lived pore [105,115], allowing for the diffusion of arginine-rich cell-penetrating peptides and their cargo.

### 4.4. Mechanisms of Resistance of Prokaryotes to LL-37

LL-37 interacts with the membrane of bacteria mostly by electrostatic interactions. The outer membrane of many Gram-negative bacteria contains LPS with lipid A, an anionic lipid forming the outer leaflet of the membrane. The transfer of aminoarabinose [116] to lipid A or its acylation [117] constitutes an alteration of the LPS which decreases the net negative charges of the membrane. Gram-positive bacteria are enveloped by a thick wall rich in teichoic acid and peptidoglycan which explains its electronegativity. Coupling of alanine via an ester bond to the chain of teichoic acid introduces the positive charge of the amino group of the amino acid [118]. The synthesis of a derivative of phosphatidylglycerol containing a residue of lysine is another mechanism developed by Gram-positive bacteria to resist to LL-37 [119]. Some bacteria secrete proteases which inactivate LL-37 [120]. In some cases the resistance to the peptide is mediated by an efflux pump driven by a proton-motive-force and which usually confers resistance to dyes or disinfectants [121]. Other bacteria like *Streptococcus pyogenes* secrete a protein which binds to and neutralizes LL-37 [122]. Inhibition is sometimes exerted at the transcriptional level [123].

### 4.5. Effect of LL-37 on Biofilms

Bacteria spend most of their life not in planktonic conditions but within a biofilm. In a first step the bacteria adhere to a support. Planktonic and adherent bacteria have a different transcriptome [124]. The cells engaged in the process of biofilm formation express enzymes involved in the synthesis and secretion of exopolysaccharides and proteins contributing to the expansion of the biofilm [125]. The expansion phase of the biofilm impairs the transport of nutrients and oxygen. This phase is paralleled by an increased number of bacteria which exacerbates the metabolic stress exerted on bacteria. This is followed by the rupture of the biofilm and the release of bacteria which recover their initial transcriptome and start to colonize the support at various other locations [126]. The development of a biofilm is very often associated with a decreased sensitivity to treatment [127,128]. The resistance to antibiotics can be secondary to interaction of the drug with the extracellular matrix which decreases its concentration in the vicinity of the bacteria [129]. Bacteria also start to express some proteins contributing to their resistance to antibiotics [130]. The polysaccharides which are major components of the biofilms are rich in negative charges and neutralize cationic antimicrobial peptides by electrostatic interactions [131,132]. However, recent results suggest that biofilms might not always be an absolute protection for the bacteria against antimicrobial peptides. BMPA-28, which is a 28-amino acid cathelicidin, also inhibited the formation of a biofilm by *Staphylococcus aureus* on intravenous catheters [133]. Overhage *et al.* [134] reported that very low concentrations of LL-37 inhibited the formation of biofilms by *Pseudomonas aeruginosa* *in vitro*. It also affected preexisting biofilms. Instillation of LL-37 also inhibited the formation of a biofilm by *Pseudomonas aeruginosa* in a model of sinusitis developed in rabbits [135]. More recently, Hell *et al.* [136] reported that infra-bactericidal concentrations of LL-37 inhibited the attachment of *Staphylococcus epidermidis* in wells of microtitre plates and the formation of a biofilm by these bacteria. Amer *et al.* [137] used a similar protocol to demonstrate that very low concentrations of LL-37 also inhibited the formation of a biofilm by *Francisella*.

## 5. Effects of LL-37 on Eukaryotic Cells

The original description of cationic peptides as antimicrobial agents put the emphasis on their deleterious effects on bacteria. The specificity of cationic antimicrobial peptides for bacterial membranes was explained by the anionic properties of bacterial surfaces [138]. The high cholesterol content of the plasma membrane of eukaryotic cells did not prevent the binding of LL-37 but blocked its penetration in the bilayer [139]. Any effect on eukaryotic cells was considered as a side-effect inasmuch as high concentrations of the peptides proved to be cytolytic on these cells [140]. It was thus mandatory to design peptides with the highest bactericidal activity at concentrations without any toxic activity on eukaryotic cells. The discovery that LL-37 was chemotactic for neutrophils, monocytes and T cells created a new bridge between innate and adaptative immunity. It was suggested that the peptide could bind to the formyl peptide receptor-like 1 (FPRL-1), a G-protein coupled receptor (GPCR) [57]. In line with this hypothesis CRAMP was chemotactic for HEK-293 cells transfected with the human or murine form of FPRL-1 confirming that these receptors mediated the response to LL-37 [57]. The activation of FPRL-1 by LL-37 was also responsible for the anti-apoptotic effect of the peptide on neutrophils [141]. The peptide promoted angiogenesis by interacting with the FPRL-1 receptor expressed by endothelial cells [142]. Two other GPCR have more recently been linked to LL-37. Zhang *et al.* [143] reported that LL-37 could selectively down-regulate the CXCR2 receptor on human neutrophils. LL-37 also elicited all the responses coupled to this receptor suggesting that it was a full agonist. Brandenburg *et al.* [144] observed that a metabotropic purinergic receptor, the P2Y_11_ receptor of glial cells, was activated by LL-37. This interaction provoked the expression of various cytokines and the activation of phosphorylation pathways. The activation of mast cells by LL-37 might also be mediated by a GPCR. Indeed, the peptide was chemotactic for these cells and stimulated their degranulation [145] and their production of prostaglandins [146]. These responses were blocked by inhibitors of GPCR signalling pathways [147]. Elssner *et al.* [148] reported that LL-37 could stimulate the release of IL-1β by LPS-primed monocytes. This response was blocked by inhibitors of P2X_7_ receptors. It was not secondary to the release of ATP since apyrase, an enzyme which catabolizes extracellular ATP, had no effect on the response to LL-37. These results suggested that the P2X_7_ receptor was a target for LL-37. This receptor is an ionotropic purinergic receptor with two transmembrane domains [149]. After activation with ATP, they form a homotrimer or even a heterotrimer with P2X_4_ receptors [150]. More recently, Tomasinsig *et al.* [151] correlated the ability of cathelicidins to activate P2X_7_ receptors with their ability to form an alpha-helix and to aggregate. LL-37 also provokes cell proliferation and wound healing [152]. It can also be the trigger for tumour proliferation [153,154,155,156]. The mechanism involved in the response is complex. It might be secondary to the activation of a still-uncharacterized GPCR responsible for the activation of a transmembrane metalloprotease which belongs to the family of ADAM [157]. This activated protease could contribute to the ectodomain shedding of ligands which might transactivate receptors for EGF (EGFR) and promote cell proliferation. 

These multiple effects of LL-37 and the diversity of plasma membrane receptors which might be affected by the peptide raised some doubts on a direct interaction between these receptors and the peptide [40,158]. For instance LL-37 might regulate P2X_7_ receptors by modifying the physico- chemical state of the membrane rather than by directly interacting with the receptor. Such a model has been proposed by Zughaier *et al.* [159] and by Di Nardo *et al.* [160] to explain the regulation of TLR4 by LL-37. This hypothesis is supported by the decrease of the anisotropy of plasma membranes of eukaryotic cells exposed to the peptide [161]. This decrease reflects a modification (increase) of the fluidity of the plasma membrane. LL-37 also modifies the cellular metabolism of phospholipids by activating phospholipase A_2_ and by inhibiting phospholipase D [161]. According to Rao [162], these effects are generally observed with membrane active peptides and are best explained by a modification of the packaging of the lipid. The LL-37 might thus regulate the cellular function by modifying the physico-chemical state of the membrane rather than by interacting with a plasma membrane receptor, a model proposed by Perregaux *et al.* [163], who suggested that the cationic peptides activate macrophages by perturbing their plasma membrane. LL-37 might also regulate the activity of P2X_7_ receptors by binding to the intracellular *C*-terminal domain of the receptor [164]. Indeed, contrary to the six other P2X receptors, P2X_7_ has a very long *C*-terminal extension through which this receptor interacts with intracellular proteins [165]. In 2004 Sandgren *et al.* reported that the peptide could cross the plasma membrane [166]. The peptide used special domains of the plasma membrane called lipid rafts. Rafts are rich in cholesterol and sphingolipids explaining why these zones are rather rigid [167]. Such a transport might explain how LL-37 interacts with an intracellular receptor. It has indeed been recently proposed that in monocytes LL-37 binds to glyceradehyde 3-phosphate dehydrogenase, an enzyme of the glycolytic pathway [168]. According to these authors, this interaction regulates the p38 MAPK and cytokine production. 

## 6. LL-37, a Cell-Penetrating Peptide

LL-37 belongs to the cell-penetrating peptide family [166,169]. These peptides have been classified into arginine-rich peptides and amphipathic peptides [170]. Penetratin, the peptide derived from the third helix of the homeodomain of the drosophile transcription factor Antennapedia without the *N*-terminal glutamate (RQIKIWFQNRRMKWKK) (peptide 43-58) [171], and the HIV1-Tat derived nonapeptide (RKKRRQRRR) [172], the first cell-penetrating peptides described, belong to the arginine-class of peptides. Similar sequences are found in the A chain of the Shiga toxin from *Shigella dysenteriae* (RFRQIQRGFR) or in the A chain of ricin from *Ricinus communis* (RTRIRYNRR) [173]. In solution, these cell-penetrating peptides adopt a random coil structure [174,175]. In a lipidic environment like membranes they adopt alpha-helical or beta-sheet secondary conformations [176,177,178]. Penetratin has a chameleon-like behaviour and modifies its conformation according to the lipid/peptide ratio or the charges on the surface. At high ratio or in the presence of low surface charge, the peptide has mostly an alpha-helical structure which transforms to beta-sheet at lower ratio or higher surface charge [179]. These peptides are internalized through two major mechanisms [180]. They might cross the plasma membrane through translocation, an energy-independent mechanism [181,182], or be internalized by endocytosis which originates at lipid rafts [183]. The internalization mechanism of the two classes of peptides differs: arginine-rich peptides seem to follow the endocytosis and vesicular pathway [184]. The intracellular location of the peptides has been a matter of debate. It was originally suggested that they had access to the nucleus [185], but this is apparently a post-fixation artefact [186] and the peptides seem to localize either in a compartment of endosomal vesicles or in the whole cytoplasm [187]. This is a very important issue when considering pharmaceutical applications of these peptides. They have indeed been considered as Trojan horses for the transmucosal or transmembrane transfer of molecules like peptides or proteins, polysaccharides, ions, nucleic acids [188]. These peptides share with LL-37 its antimicrobial or antifungal properties [189,190]. Conversely Lande *et al.* [191] showed that LL-37 was able to form a complex with human DNA and to transfer the nucleic acid to the endosomes. Zhang *et al.* [192] confirmed that LL-37 could mediate the cell delivery of oligonucleotides. Recently, Hurtado and Au Peh [193] reported that LL-37 promoted the rapid delivery of bacterial CpG motifs to human B lymphocytes and pDC. This response was independent of the amphipathic and bactericidal properties of LL-37 since GL-37, a scrambled analog, could reproduce the response to LL-37. These results suggested that the combination of CpG with LL-37 was a very efficient combination to stimulate TLR9. This intracellular receptor is expressed by macrophages, conventional dendritic cells and pDC and B lymphocytes. Its activation triggers a cascade of events which greatly improves adaptative immunity [194]. Indeed, stimulated pDC secrete type I IFN [195] which activates cytotoxic and helper T cells [196] and NK cells [197]. Activated cDC stimulate pDC and NK cells [198]. They also promote the differentiation of Th0 lymphocytes to the Th1 phenotype [199]. The activated B lymphocytes proliferate and secrete immunoglobulins of the M or G type rather than allergic immunoglobins [200]. The injection of a mixture of CpGs and LL-37 to mice which had been treated with ovarian cancer cells enhanced the survival of the animals when compared to treatment with either CpG or LL-37 alone [201]. Tumours grow faster in cathelicidin knockout mice. NK cells from these mice had impaired cytotoxic activity toward tumour cells [202]. These promising results suggest that LL-37 or some analogs might be very potent adjuvants [203,204]. They should however be confirmed inasmuch as Nijnik *et al.* [205] reported that LL-37 inhibited the immune responses in response to IFN-γ. 

## 7. Conclusions and Perspectives

LL-37 was originally described as an antimicrobial peptide. The development of analogs of the peptide with increased resistance to proteases and less cytotoxicity on eukaryotic cells, or the synthesis of molecules mimicking LL-37 is a promising strategy to eradicate multiresistant bacteria [206]. Low, non-cytotoxic concentrations of LL-37 have now proven to be also effective on eukaryotic cells. The topic application of the peptide on skin wounds or bedsores should promote wound healing. But the immunomodulatory properties of the peptide secondary to its regulation of intracellular receptors probably offer the most fascinating perspectives. 

## Abbreviations

cDC: conventional dendritic cells;
CRAMP: cathelicidin related antimicrobial peptide;
EFGR: epidermal growth factor receptor;
FPRL-1: formyl peptide receptor-like 1;
GPCR: G-protein coupled receptor;
IFN: interferon;
LPS: lipopolysaccharides;
NK cells: natural killer cells;
pDC: plasmacytoid dendritic cells;
PE: phosphatidylethanolamine;
PG: phosphatidylglycerol;
PS: phosphatidylserine;
TLR: toll-like receptor.
